# Using a tele-behavioral health rapid intake model to address high demand for psychotherapy at an academic medical center during COVID-19

**DOI:** 10.3389/fpsyt.2022.989838

**Published:** 2022-12-22

**Authors:** Kristina McMahan, Karli M. Martin, Melissa J. Greenfield, Pamela Hay, Madison Bates Redwine, Rachel Fargason, Kristine Lokken

**Affiliations:** ^1^Department of Psychiatry and Behavioral Neurobiology, University of Alabama at Birmingham, Birmingham, AL, United States; ^2^Alabama Psychiatry, Helena, AL, United States

**Keywords:** COVID-19, pandemic, mental health, behavioral health, telehealth

## Abstract

**Background:**

Long wait times for mental health appointments have been a chronic dilemma for academic medical centers. This problem intensified worldwide with the onset of the COVID-19 pandemic. Approximately 70% of mental health services experienced pandemic-related disruption in care provision, while simultaneously experiencing a substantial increase in patient demand. Wait times for mental health appointments also increased, varying across populations from 3 to 18 months. As prolonged wait time is positively associated with severity of psychiatric symptoms and negative outcomes, the authors implemented a novel rapid intake telemedicine clinic model to shorten wait time and increase patient access to psychological care at an academic medical center.

**Methods:**

To address an overwhelming influx of mental health referrals and a growing wait-time-until-first appointment at an academic medical center serving as a lone safety net hospital during the COVID-19 pandemic, a 5-provider Psychology Rapid Intake Team was established using a hybrid of telehealth and in-person appointments based on patient preference. Data on new patient volumes, wait time for 1st appointment, and wait time to begin therapeutic intervention were compared during the same calendar 3-month period immediately prior to and following implementation of the rapid intake clinic.

**Results:**

A paired-samples *t*-test was conducted to compare new patient volumes pre- vs. post- intervention. Results revealed a significant increase in the number of new patients the providers were able to accommodate in the post-implementation (M = 62.00, SD = 7.21) compared to the pre-implementation (M = 31.00, SD = 2.61) condition; *t*(2) = −8.60, *p* < 0.05. There was a significant decrease in the average wait times for 1st appointment post-implementation (M = 24.99, SD = 2.38) compared to the pre-implementation (M = 37.32, SD = 1.47) condition; *t*(2) = 5.56, *p* < 0.05. In addition, days to begin therapeutic intervention decreased dramatically (394%) from the pre- (M = 142.50) to post-implementation (M = 28.84) period.

**Conclusion:**

The COVID-19 pandemic strained a mental healthcare system which led to increasingly long wait times for intake appointments and delayed psychotherapy interventions. The Psychology Rapid Intake Team initiative served to improve access, reduce patient risk related to prolonged wait times, and accelerated patient engagement with psychotherapy services. The model can serve as a unique, sustainable infrastructure for behavioral health delivery for low acuity mental health problems in large health care systems.

## 1 Introduction

Timely access to mental health services is a prevailing issue worldwide, and in particular among the southern US states with Mississippi (#47), Georgia (#48), Florida (#49), and Alabama (#50) ranking the lowest among states in access to mental health care (1). Preceding the pandemic, over two-thirds of primary care providers (PCPs) reported difficulty accessing mental health care for their patients (2). A 2014 review of national electronic medical records examined wait times from Family Practice physician referrals to several different specialists (e.g., surgery, plastics, ENT, rheumatology, gastroenterology, cardiology, urology, orthopedics, dermatology, and psychiatry). Findings revealed the median wait time for psychiatry appointments was second only to gastroenterology, at 73 days, and the 75th percentile wait time of 231 days for Psychiatry appointments was nearly double in comparison to other specialty services (3).

Delayed access to appropriate mental health services has been further amplified by the coronavirus pandemic starting in 2020 due to unprecedented ensuing social stressors. A recent scientific brief released by the World Health Organization (4) indicated the global prevalence of major depressive disorder (MDD) and anxiety disorders increased by 27.6 and 25.6%, respectively, in the first year after the pandemic began (4). Additionally, there is a trend for increases in all neuropsychiatric disorders post-pandemic, further increasing demand for mental health services, including psychotherapy. Psychological interventions are proven to be effective in treating mental health disorders and have shown effectiveness in preventing or reducing pandemic-related mental health problems (4). However, 62% of psychologists reported increased post-pandemic referrals leading to long wait times and waitlists for psychotherapy services (5).

Wait times are an important measure of access to care. When access to care is delayed, (a) there is reduced efficiency of the agency (6); (b) patient satisfaction declines (7); (c) the likelihood of patient no-show to appointment is increased (6), when no-show rates for new patients are already as high 50% for mental health providers (7); and (d) health and mental health outcomes are worse (8). Reducing wait times for mental health services can serve to increase patient stability, decrease relapse, decrease crisis-related hospitalizations, and decrease attempted and completed suicide (6).

To protect provider health and reduce patient exposure risks, over 70% of countries adopted telehealth models to overcome disruptions in medical services during the height of the pandemic (4). Although disparities may exist in the uptake of telehealth services, virtual mental health platforms have offered a unique way to bridge gaps in the provision of services and further scale mental healthcare (9).

The purpose of this report is to describe the implementation of a tele-behavioral health rapid intake model to address high demand for psychotherapy resulting in long wait times for services at a Southeastern US academic medical center during COVID-19 (10, 11). A primarily telehealth platform was utilized to leverage access; however, patients were offered the option to choose either telehealth or in-person sessions for their rapid intake appointment and for ongoing therapy once matched to an interventionist. Of note, >90% of patients chose the telehealth option for their rapid intake session. This report may be valuable in informing academic medical centers striving to develop clinic models to improve patient access and clinic efficiencies, given the global mental health burden following COVID-19.

## 2 Materials and methods

### 2.1 Description of the problem

As with other mental healthcare systems, there was increased demand for psychotherapy following the COVID-19 pandemic for the Department of Psychiatry at a Southeastern US academic medical center. Based on data indicating the doubling of community calls to the access center and a new system wide provider ordering system, it was estimated that general psychotherapy referrals had increased six-fold from Fall of 2019 to the Fall of 2020, following the start of the pandemic. This resulted in long patient wait times, with accompanying problems of higher no-show rates, increased patient distress and risk, and faculty overbooking/burnout. A bottleneck emerged stymieing access to psychotherapy primarily due to inappropriate matching of new patient referrals or patients scheduled with providers with psychiatric needs outside of the realm of the provider’s area of expertise. Stakeholders and hospital administration were consulted and agreed to the hire of additional psychology faculty members to off-set the increased need in mental health services; however, hiring psychology faculty generally requires between 9 and 18 months to fully onboard licensed, credentialed providers. It was determined other immediate steps needed to be taken.

Data from July 2021 indicated wait-time-until first psychotherapy intake was approaching a mean of 5 months (range 92–212 days), thus impacting timely access to needed psychological care. The no-show rate for new appointments for patients referred by non-psychiatry providers approached 50%. Many patients presenting for care required further referrals to more appropriate providers, such as a psychopharmacologist, despite their long wait time for services. Such patient-provider mismatch was frustrating both to needful patients and beleaguered frontline clinic staff who took the brunt of patient frustration, as did the overburdened but under-utilized psychotherapy providers.

In August of 2021, this interdisciplinary team from the Department of Psychiatry, collaborated in a quality improvement (QI) project to develop an effective rapid intake model to improve access, address potential risk associated with delayed psychological intervention, and accelerate patient engagement with appropriate and empirically validated psychotherapy services. The principle aims of this QI project were to increase access to more immediately assess risk of suicide for patients seeking psychotherapy. We hypothesized that providing multiple rapid intake-only appointments per week (approximately 20) with highly trained psychodiagnosticians (e.g., Clinical Psychologists) would not only serve more patients per week, but also increase efficiencies in our agency’s systems. The rapid intake appointments could provide a mechanism to absorb the well-documented no-show rates associated with initial psychological intakes, thus reducing downstream no-show rates for limited, hence valuable, long-term psychotherapy provider intake slots. The rapid intake appointments could also provide a mechanism to address the bottleneck associated with patients reluctant to begin therapy or inappropriate referrals. Our combined experience suggested that conducting initial intakes with ambivalent patients in a rapid intake clinic model could serve to either increase that patient’s understanding of the usefulness of psychotherapy through education, thus increasing patient engagement with long-term psychotherapy providers, or could connect those uninterested in psychotherapy to more appropriate resources. It was hypothesized that quickly identifying those patients better served by other university and community services [e.g., free psychotherapy services for university employees and students, state-funded Community Psychiatry Program services for seriously mentally ill (SMI), social service agencies for housing and food assistance, or psychopharmacology providers] would further reduce downstream inefficiencies. Identifying these patients early and connecting them with more appropriate resources would allow full utilization of psychotherapy provider appointments for well-matched patients. We also hypothesized that getting new patients in rapidly for risk assessment to identify safety issues, suicidality, and symptoms of SMI would ultimately reduce risk. The secondary arm of the intervention model was to employ a Match System to funnel appropriate patients to the most appropriate psychotherapist or mental health provider. We predicted this model would improve provider satisfaction, and likely lead to improved patient engagement and outcomes. Additionally, as a subset of patients seen by the Psychology service had to then be referred on for psychopharmacology management resulting in additional long waits, an innovative model to integrate psychology and psychiatry services was included as a pilot within the larger project.

As proposed outcome measures, baseline levels of new patient volumes, wait time for 1st appointment, and wait time to begin therapeutic intervention were reviewed for later comparison following implementation of the rapid intake clinic.

### 2.2 Specific aims

#### 2.2.1 Primary aim 1

The key aim of this initiative was to improve access to Psychotherapy Services.

• Benchmark: To address primary aim 1, new patient volume was identified as the outcome measure and compared between the pre- and post-intervention. Our goal was to increase access by 10%.

#### 2.2.2 Primary aim 2

Primary aim 2 was to reduce waitlisted patient morbidity and suicide risk.

• Benchmark: To address primary aim 2, wait time for 1st appointment and wait time to begin therapeutic intervention were identified as the outcome measures. We set the goal of providing the first intake appointment with a full diagnostic interview and risk assessment within 10–14 days from referral.

Secondary aims of the intervention were measured using quantitative and qualitative data gathered from an employee survey at baseline (beginning of the intervention in September of 2021) and again in follow-up in June 2022 (10-month post-intervention).

#### 2.2.3 Secondary aim 1

We aimed to initiate a Patient-Provider Match System with the goal of “right patient-right provider.”

• Benchmark: Quantitative and qualitative data on an employee survey administered to psychology faculty before and after the initiative would show improvements in psychologist reported fit of patients to expertise and effectiveness in role.

#### 2.2.4 Secondary aim 2

We aimed to improve employee satisfaction as a measure of the Success/Failure of the Rapid Intake Model.

• Benchmark: A qualitative employee satisfaction survey provided at baseline and 10-month post-intervention to psychology faculty, Intradepartmental Referring Providers, and Department of Psychiatry scheduling and front desk staff would show increased employee satisfaction post-intervention.

#### 2.2.5 Secondary aim 3

We evaluated proof of concept for a Psychology + Psychiatry interdisciplinary Intake model.

Benchmark: The weekly Psychologist + Psychiatry intake clinic would demonstrate utility and feasibility of the cross disciplinary model by: (a) staying full with appropriate referrals who benefited from both providers’ skillsets; (b) providing appropriate and evidence based initial care without patient complaint or negative outcome; (c) further improving the efficiency of the Psychology Rapid Intake Team (PRIT) clinic by drawing off less appropriate referrals; (d) demonstrate that a psychology trainee and psychiatry attending could work collaboratively with a single patient.

### 2.3 Setting, context, and description of resources

The project was based in the Department of Psychiatry at a Southeastern academic medical center serving as a lone safety net hospital during the COVID-19 pandemic where approximately 55,000 outpatient clinic encounters occur per year. Pre-intervention (March 2021–May 2021), psychology faculty included 11 Ph.D. level psychologists; however, only 3 full time equivalents (FTE) are devoted to general psychotherapy services. As a group, psychology faculty provide therapy intervention for specialty clinics including Functional Neurological Disorder, Behavioral Sleep Medicine, Integrated Behavioral Medicine Service (iBeMS), Addictions, LGBTQ-related needs, Serious Mental Illness through the Community Psychiatry Program, Trauma-Related Disorders, and neuropsychological evaluation for psychiatric inpatients, transplant candidates, post-COVID persistent cognitive issues, Alzheimer’s, and Dementia Related Disorders (ADRD) and ADHD. Referrals for these specialty services also increased during the pandemic and the need was met with current systems.

Both pre- and post-intervention, community and other external organizations providing psychological services were difficult to access because of extensive wait lists, non-centralized referral sources, pandemic shutdowns, and staffing shortages. Psychotherapy services in other University Medical Center departments discontinued taking external psychotherapy referrals due to high internal departmental needs. The Department of Psychiatry became the sole referral source for general and specialty psychotherapy for the very large University Medical Center, with referrals including those with complex trauma and other difficult to treat characteristics. Therapy referrals to psychology services were ongoing with demand for a full range of mental health and neuropsychiatric diagnostic services.

### 2.4 Intervention

#### 2.4.1 Development of a psychology rapid intake team (PRIT)

**FIGURE 1:**
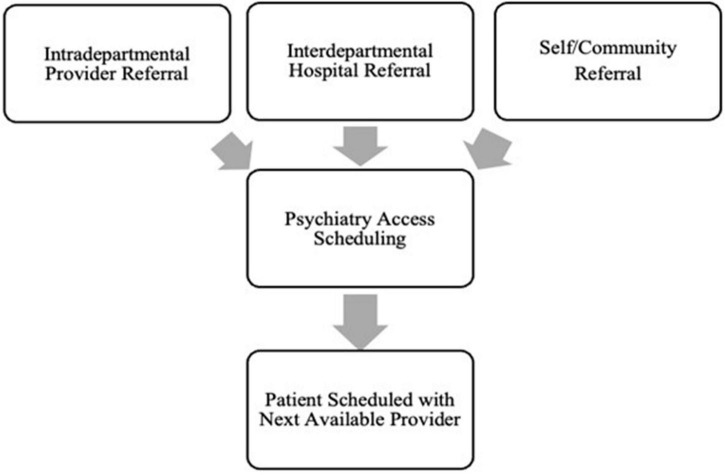
Previous appointment system.

**FIGURE 2:**
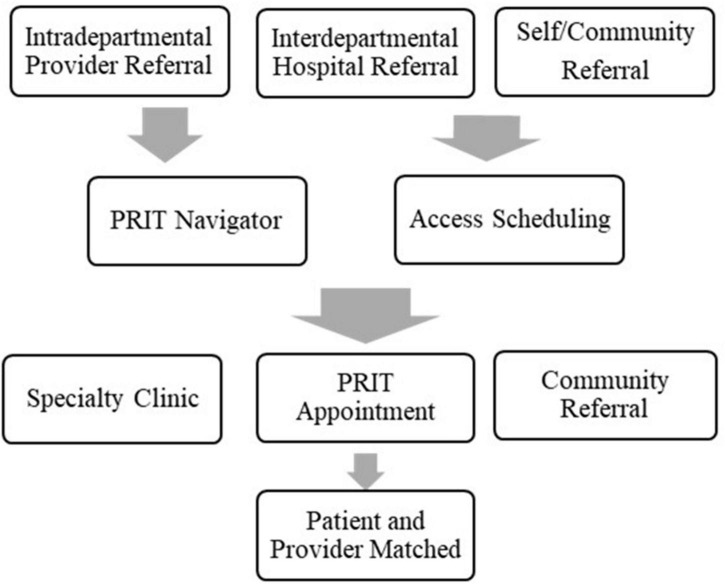
Psychology rapid intake team appointment system.

To address need, a 5-provider Psychology Rapid Intake Team (PRIT) was conceptualized and formed using a hybrid of telehealth and in-person appointments based on patient preference. PRIT initially consisted of five providers, with each dedicating one 4-h clinic block for conducting psychotherapy intakes. This resulted in 20 new intake appointments per week (∼80 per month). See Figures 1, 2 for a comparison of the previous system with PRIT system.

With the departmental chair approval and waiver from the IRB due to the quality focus of this project, an interdisciplinary steering committee for PRIT consisting of the Vice Chair of Clinical Affairs, the Director of Ambulatory Psychiatry, the Administrative Manager of Outpatient Clinics, and the Chief Psychologist in the Department of Psychiatry was formed to provide oversight on the project. The team met monthly to review and refine the plans. Goals were: (1) capitalize on telehealth given the ongoing nature of the pandemic, clinic space limitations, and convenience to patients; (2) gain control of waitlist to reduce wait times; (3) provide opportunity to administer empirically validated therapies sooner; (4) match appropriate patients with the appropriate resources and providers; (5) reduce no show rates and loss of valuable psychotherapy appointments to futile/ineffective endeavors for patients that are not well-matched for the psychologist’s skillset. The PRIT Steering Committee tracked weekly and monthly data reflecting key benchmarks and outcome data, including: (1) number of new intakes; (2) time to first appointment; (3) time to assigned provider; and (4) no show rates. New referrals continued to be accepted in an ongoing manner. To address the existing backlog of appointments, patients who had been waiting 5–7 months for appointments were scheduled to earlier PRIT appointments.

The transformation of the psychotherapy referral mechanism comprised of four PDSA (plan-do-study-act) cycles over a 1-year period, each 3 months in length. For 1 month prior and during the first 2 months of implementation of the PRIT model (August to October of 2021) the plan-do part of the model was enacted. Five PRIT providers were chosen based on availability, skill, and agreement. The PRIT providers were highly qualified and well-trained psychology faculty (3) or Psychology Post-doctoral fellows (2) supervised by psychology faculty. All PRIT providers had completed all necessary training to obtain a Ph.D. or Psy.D. in Clinical Psychology, with ample clinical experience in psychodiagnostic assessment. An armamentarium of psychological resources was curated for PRIT, including a structured psychology intake, measurement-based care questionnaires, a comprehensive community-based psychology referral resource list organized by psychodiagnostic need, and a detailed internal provider specialty list. The PRIT team met regularly and shared ideas, concerns, and strategies to help inform the model. Next, the front desk staff and the Access Scheduling Team were trained on the new model. These trainings consisted of several initial meetings with front desk staff and the Access Scheduling Team, followed by regular detailed emails outlining new systems and any changes to the systems as the PDSA period progressed. In September of 2021, prior to the full implementation of the PRIT model, an online survey was created and distributed among psychology faculty, Support Staff (front desk clinic staff and the Psychiatry Access Scheduling Team), and departmental referring providers (Nurse Practitioners, Psychiatry Residents, Psychiatrist faculty) to garner current climate, morale, and effectiveness of current systems for psychotherapy referrals.

Initially, as with any system shift, the change proved to be very difficult and was met with skepticism and resistance. Through an iterative process of continual quality improvement, PRIT systems, workflow, roles, and boundaries were discussed and refined. The PRIT team continued to optimize workflow, define goals of intake, and the PRIT team identified a psychology faculty “navigator” to assist with scheduling appropriate referrals and disposition. Implementation of mechanisms to encourage referring provider-to-patient communication and reduce referrals not wanted by the patient to PRIT included: (1) broad distribution of the community referral resource list; (2) changing the University-wide Psychiatry scheduling order to include “patient is aware of the referral, agrees to treatment by Psychiatry, and does not currently have a therapist.”

Another iterative improvement to processes occurred when the PRIT team noticed that a subset of scheduled PRIT intake patients who had requested therapy also had a clear imminent need for a psychiatric provider. The group reviewed methods to identify these patients at the time the appointment was scheduled and developed a model to address this need. The pilot model of an intake dyad of psychiatrist and psychology post-doctoral fellow to simultaneously address medication management and therapy needs was conceived and tested as a within study feasibility project. The group instructed the access team to channel to the psychology/psychiatry intake dyad all patients who requested therapy but also met any of the following criterion: (a) patient requested therapy and medications (or alternatively, was unsure what they needed); (b) patient lacked a psychiatric provider but was on multiple psychopharmacology agents; (c) patient had a serious mental illness, history of psychiatric hospitalizations, semi-urgent need for care or high distress levels. Joint intake interviews involved a psychiatry attending and psychology post-doc PRIT team member and allowed all the patient’s needs to be immediately addressed. The trainee-attending model was selected as the most cost-effective as insurance would only reimburse for one provider’s service on the day of service.

At the same time, a new departmental model utilizing psychology trainee clinics with in-session supervision by psychology faculty was implemented in some of the existing psychotherapy clinics. These trainee clinic models further scaled psychotherapy services three-fold during the clinic block times. For example, where traditionally a psychology faculty member could see 4 psychotherapy patients in 4 h, three trainees per 4-h block would allow the supervising psychology faculty member to provide services and bill for >16 min on up to 12 psychotherapy patients in the same 4-h block.

During the second PDSA cycle, months 4–6 (November 2021–January 2022), vast increases in intradepartmental, university-wide interdepartmental, and community referrals were seen. PRIT providers were quickly booked out 3–4 months vs. 10–14 days, disregarding the spirit of the “rapid” in Psychology Rapid Intake Team. The need for psychotherapy services was unprecedented and increasing, possibly in relation to the aftermath of the Delta Variant of the COVID-19 virus surge. Our Psychiatry Access team received > 150 referrals for psychotherapy services from University hospital interdepartmental referral sources alone in the month of November, but there were only 70 PRIT appointment slots, so patients were scheduled much farther out than anticipated. To prevent another overload of the system with accompanying dangers and inefficiencies, the PRIT Steering Committee along with hospital leadership consent, made the difficult decision to temporarily limit future general psychotherapy referrals to only patients with contracted insurance plans. Uninsured and underinsured continued to be treated at the department’s community mental health center. Requests were sent to intradepartmental, interdepartmental, and community referrals to only refer patients with contracted insurance plans. Referring providers were encouraged to consult the curated community referral resource list. This reduced the number of referrals by approximately 20–40% and time to first PRIT appointment returned to 10–14 days by February of 2022.

During the third PDSA period, months 7–9 (February–April 2022), our model stabilized, workflow could be studied, and refinements were made. Seasoned PRIT providers were able to accommodate the initial intake in 30-min vs. 60 min so additional appointment slots were added to existing clinics. By the end of April 2022, we were pleased to be able to re-open PRIT appointments to *all* intradepartmental referrals, regardless of insurance contract.

We are now in the of the fourth PDSA period, months 10–12 (May–July 2022) and are carefully thinking of minor changes to simplify, streamline, and scale our services with PRIT 2.0, with focus on implementing patient reported outcome (PRO) measures, additional collaborative care models, better patient to therapist matching, warm hand-off to community based referrals that can better serve patient needs, and seeking approval for the addition of a sliding-scale therapy clinic for those with Medicaid/limited resources. Our long-term goal is to accommodate *all* appropriate referrals for general psychotherapy.

## 3 Results

### 3.1 Statistical analyses

The formation of the Psychology Rapid Intake Team (PRIT) comprised of four PDSA (plan-do-study-act) cycles over a 1-year period, each three months in length. Data on new patient volumes, wait time for 1st appointment, wait time to begin therapeutic intervention, and no-show rates were collected using the university’s data analytics platform and PRIT Navigator tracking methods. The data analytics platform allows appointment and provider data to be recorded in real time, for retrospective analysis. To ensure control over seasonal variability in referrals, data was compared during the same calendar 3-month period immediately prior to and following implementation of the rapid intake clinic.

Queries for data of interest were run based on date of intervention (pre vs. post intervention time points) and psychotherapy provider. Data were then codified and collated using statistical analysis software. A series of paired samples *t*-tests was used to compare data from the pre-intervention period to the post-intervention period. Data for secondary aim 1 and 2 were gathered from an employee survey at baseline (beginning of the intervention in September of 2021) and again in follow-up in June 2022 (10-month post-intervention). Data from the employee survey was codified and collated using statistical analysis software and a series of paired samples *t*-tests were run to compare survey data from time 1 to time 2.

**FIGURE 3:**
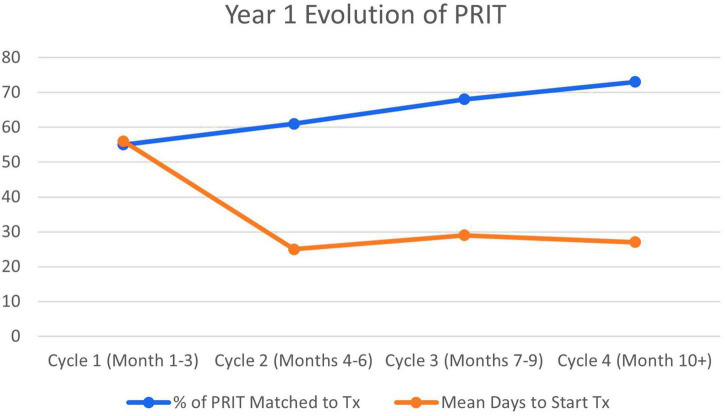
Percentage of psychology rapid intake team (PRIT) referrals matched for treatment and average days for patients to start treatment. Tx, treatment.

Figure 3 shows an evolution of the intervention during the first year of the project. Although system changes were met with many challenges in months 1–2, month 3 seemed to be a “Honeymoon Period,” where potential benefits of the PRIT systems became apparent. There was ample patient access with the five PRIT providers, and the new system allowed the rebook of several already-scheduled new patients, booked as far out as July of 2022 (1 year wait time) by the former system. These rebooked patients were provided with newly created PRIT appointments in September and October of 2021. Overall wait times for new appointments for psychotherapy were further reduced and as outlined in the diagram, time between PRIT appointment and match to long-term therapist was reduced to <1 month (17 days) by month 4 of the intervention (November 2021).

The establishment of trainee clinic models, beginning in month 6 (January 2022) allowed our PRIT team to refer more patients, more quickly for long-term therapy. Mean wait time for patients to be seen in the trainee clinics was less than 2-weeks following PRIT intake and interested patients were guaranteed weekly therapy appointments in a structured, highly supervised therapeutic setting using empirically valid therapies. This further reduced the overall wait time between PRIT appointment and match to long-term therapist from a mean of 56 days for months 1–3 to a mean of 25 days for months 4–6, a mean of 29 days months 7–9, and a mean of 27 days for months 10–11. As wait time to intervention decreased, the number of patients seen and matched to psychotherapy providers in our department simultaneously increased from 55% in Cycle 1 to 73% in Cycle 4.

The PRIT appointments fielded a high number of no-shows for appointments, with monthly averages ranging from 7 to 36%. At the same time, no-show rates for psychotherapy providers declined (from 52% pre-intervention to 18% post-intervention), suggesting the rapid intake appointments served to absorb downstream no-show rates for long-term psychotherapy provider intake slots. A surprising additional finding was that no show rates were higher for those offered an appointment < 1 week from referral and for those offered an appointment farther into the future (>1 month from referral). This was discussed among team members who observed that there may be a “sweet spot” time interval to rapid intake appointment. We hypothesized that if the appointment was offered too soon, the patient possibly did not value it and may have assumed they could get an appointment whenever desired, so let other competing time demands take precedence. Alternatively, if the appointment was scheduled too far into the future, the initial crises could resolve, or patients may forget about the appointment date and time. This information was deemed critical and the original goal of PRIT appointments between days 10 and 14 of contact was deemed essential to the efficiency of the intervention.

Education and training efforts for staff and referring providers also seemed to provide benefit, with numbers of inappropriate referrals to the PRIT clinic declining over time (23% in Cycle 1 vs. 4% in Cycle 3).

**FIGURE 4:**
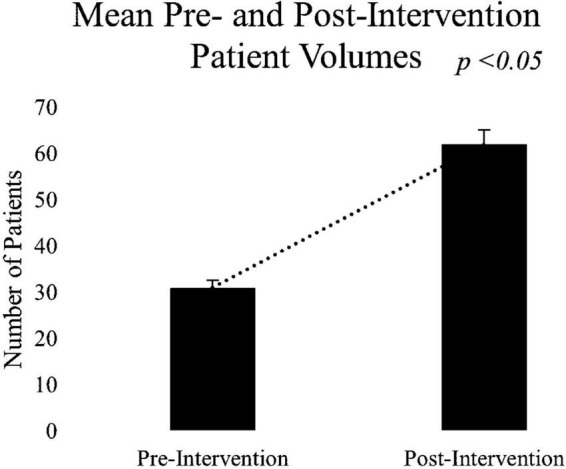
Number of new patients that providers accommodated in pre-and post-intervention.

To examine primary aim 1, to improve access to Psychotherapy Services, a paired-samples *t*-test was conducted to compare new patient volumes pre- (March-May 2021) vs. post- intervention (March-May 2022). Results revealed a significant increase in the number of new patients that providers were able to accommodate in the post-implementation (M = 62.00, SD = 7.21) compared to the pre-implementation (M = 31.00, SD = 2.61) condition; *t*(2) = −8.60, *p* < 0.05. Results indicated the target goal of increasing access by 10% was far exceeded (see Figure 4), with patient volumes increasing by 200%.

**FIGURE 5:**
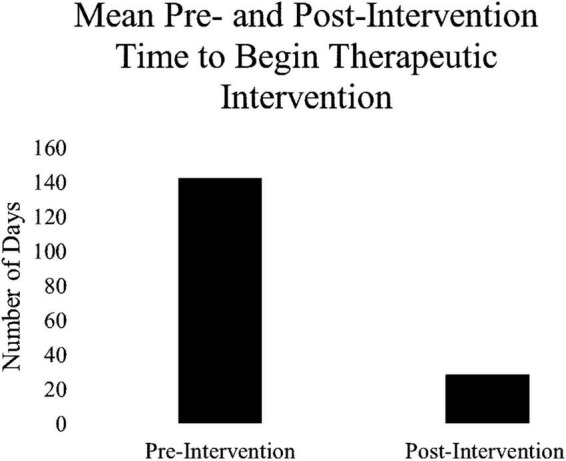
Average number of days waited before beginning therapeutic intervention.

To examine the primary aim 2, to reduce patient morbidity and suicide risk, data for wait time for 1st appointment and data for wait time to begin therapeutic intervention were identified as the outcome measures. Results of a paired-samples *t*-test indicated there was a significant decrease in the average wait times for 1st appointment post-implementation (M = 24.99, SD = 2.38) compared to the pre-implementation (M = 37.32, SD = 1.47) condition; *t*(2) = 5.56, *p* < 0.05. In addition, days to begin therapeutic intervention decreased dramatically (394%) from the pre- (M = 142.50) to post-implementation (M = 28.84) period (see Figure 5). Although trending in the right direction, the goal of providing the first intake appointment within 10–14 days from referral was not consistently met.

Secondary aims of the intervention were measured using data gathered from an employee survey at baseline (beginning of the intervention in September of 2021) and again in follow-up in June 2022 (10-months post-intervention). Secondary aim 1 was to initiate a Patient to Provider Match System using a “right patient-right provider” model. Quantitative and qualitative data on psychology faculty satisfaction were used as outcome measures for this aim. Quantitative data was analyzed; however, there was no positive or negative change on pre- and post-interventions measures for psychologists’ report of patient-fit to provider-expertise, effectiveness of role, or satisfaction with schedule. However, qualitative data showed positive responses specifically related to the Rapid Intake Model (see Table 1).

**TABLE 1:** General employee reported themes about the impact of the rapid intake model.

Psychologists
**Patient-fit**
1) Takes the burden of intensive intakes that may not be the right fit off of provider. Helps match fit.
2) Screens referrals to make sure they are appropriate for psychotherapy; able to funnel referrals to best fit with the most appropriate provider; keeps non-psychologists from trying to figure out who is most appropriate.
3) It’s helpful to not have to take on patients that don’t match my expertise.
**Referrals**
1) Reaching out for chart review works well.
2) Referring providers tend to refer directly to me and Psychiatry Access schedules those patients into my clinic.

**Administration**

**Time**
1) The patients are able to get quicker appointments with a provider for therapy.
2) Allowing patients the opportunity to be seen in manageable time.
3) The model was able to get patients in right away in some instances.

**Medical doctors, nurse practitioners, residents**

**Time**
1) Patients can get established with the department quickly, which is helpful in semi-crisis situations.
2) I like that you are able to see patients relatively soon after we put in the consult.
3) Rapid disposition of therapy patients.
**Patient-fit**
1) I like that you are able to select their provider based on your interview and who would be the best fit.
2) Linking patient to the appropriate provider; ability to triage patient care according to acuity.
3) From what I can tell, using this model has allowed patients to be placed with an appropriate therapist based on patients’ needs.
**Referral**
1) Ease of referral and certainty patient will get a therapy referral.

Question: What are some ways that the psychology rapid intake team model is useful?

Secondary aim 2 was to improve employee satisfaction as a measure to Examine Success/Failure of the Rapid Intake Model. Approximately 93% of the 30 Department of Psychiatry employees who completed the survey reported the model was useful. Of that, 100% of psychology faculty, 100% of psychiatry (MD) faculty/nurse practitioners/psychiatry residents, and 60% of administration staff reported the model as useful. The qualitative employee satisfaction survey provided at baseline and 10-month post-intervention was reviewed. As shown in Table 1, comments indicated predominantly positive results regarding better referral processes with PRIT. This included more appropriate wait-times to see patients, improved ability to match patients with appropriate providers, and overall responses indicating smoother referral process and favorable adoption of the PRIT model by psychology faculty, referring providers, and staff.

Secondary aim 3 was to evaluate proof of concept with a Psychology + Psychiatry interdisciplinary Intake model. Utility and feasibility of the cross disciplinary model was achieved, as determined by 0% unfilled slots, no patient complaints or reported negative outcomes associated with this interdisciplinary intake model, and collaborative relationship between the psychology trainee and psychiatry attending. A skilled departmental advanced practice practitioner from the department was recruited to manage follow-up psychopharmacology needs on an ongoing basis.

## 4 Discussion

As with other mental healthcare systems, increased need for mental health care caused strain on existing provider systems for the Department of Psychiatry at a Southeastern US academic medical center. A quality improvement project was developed by this team with primary goals of improving patient access to psychotherapy services and reducing risk known to be associated with delayed psychiatric care. By identifying stakeholders, using continual quality improvement principles with plan-do-study-act (PDSA) cycles, and capitalizing on a teamwork approach, access to psychological care was transformed in a rapid manner without adding provider resources. Centers can transform processes to improve access to psychiatric care while simultaneously improving provider and staff satisfaction.

Over a 12-month period, the development of the Psychology Rapid Intake Team (PRIT) comprised of four PDSA (plan-do-study-act) cycles, with each cycle 3 months in duration. Results of the intervention indicated improved access to Psychotherapy Services from pre- to post-intervention, with expectations far exceeding the original goal. Additionally, wait times for 1st appointment and time to begin therapeutic intervention were dramatically improved. The goal of providing the first intake appointment within 10–14 days from referral was not consistently met and refinements to the model are continuing. Upcoming improvement initiatives to consistently meet the 10–14-day psychotherapy intake appointment goal for the PRIT model are to: increase the number of PRIT appointments offered by adding additional PRIT providers, protecting additional time for appointments on current PRIT provider schedules, and decreasing PRIT appointment types from 60 to 30 min for true risk assessment and disposition. The team is acutely aware that if the demand for appointments exceeds the supply, the model will no longer work.

No show rates remained high for PRIT providers; however, the model reduced no show rates for long-term therapy providers, thus increasing efficiency within the system. Importantly, wait lists were eliminated for general psychotherapy interventions early on (month 3) in the process.

Secondary aims of initiating a Patient to Provider Match System “right patient-right provider,” improving employee satisfaction, and evaluating proof of concept with a Psychology + Psychiatry interdisciplinary Intake model to leverage psychiatric expertise in an innovative way, were also met; however, it is noted that there was no positive or negative change on pre- and post-interventions measures for psychologists’ report of patient-fit to provider-expertise, effectiveness of role, or satisfaction with schedule despite qualitative data showing positive responses specifically related to the Rapid Intake Model. This finding indicates that further exploration should be done to address provider satisfaction, beyond system efficiencies. Provider wellness programs and mentoring are of utmost importance for mental health providers.

A limitation of the intervention included the confound of temporarily limiting general psychotherapy referrals to only patients with contracted insurance plans during months 6–9 of the intervention. We believe that this difficult decision was needed to further refine systems of care and anticipate future ability to accommodate all appropriate referrals for general psychotherapy. Uninsured and underinsured continued to be treated at the department’s community mental health center and as an alternative to PRIT, patients without contracted insurance plans were referred directly to our specialty clinics in the department or to appropriate community providers. High risk patient continued to be seen on an as-needed, *pro bono* basis as necessary. Additional limitations were potential confounding factors that affect system changes beyond those that were measured, such as integrated behavioral health pilot projects started in temporal proximity in 2 primary care clinics, expanding trainee clinics, and other novel tele-behavioral health options developed during a pandemic. As such, it is difficult to directly attribute success to any one strategy.

The team is examining the lessons learned and already working toward further refinements of the PRIT intervention. Future goals are to increase group therapy, gathering pre- and post-psychotherapy intervention patient reported outcome (PRO) measures to examine success of specific therapy interventions, examining measures of patient engagement, and further understanding downstream effects of rapid access to psychiatric care in a health system by examining psychiatric hospitalizations and ER presentation data.

Multiple gains in clinical efficiencies are possible when effort is made to reimagine traditional approaches to national mental healthcare models. The authors believe the PRIT model can serve as a unique infrastructure for behavioral health delivery to patients with low acuity mental health needs in large health care systems. This report may be valuable in informing academic medical centers striving to develop clinic models to improve patient access and clinic efficiencies and future studies should examine whether this model could be replicated.

## Data availability statement

The raw data supporting the conclusions of this article will be made available by the authors, without undue reservation.

## Ethics statement

Ethical review and approval was not required for the study on human participants in accordance with the local legislation and institutional requirements. Written informed consent for participation was not required for this study in accordance with the national legislation and the institutional requirements.

## Author contributions

RF, KMM, and KL contributed to conception and design of the study. KM organized the database. KM and KL performed the statistical analysis. KL wrote the first draft of the manuscript. All authors contributed to sections of the manuscript, manuscript revision, read, and approved the submitted version. KM helped formulate revisions and maintained communication among all authors for edits, suggestions, and revisions.
